# Effects of UV-C Irradiation on Postharvest Quality, Bioactive Compounds, and Antioxidant Activity of Seriguela Fruit (*Spondias purpurea* L.) During Ambient Storage

**DOI:** 10.3390/foods15091604

**Published:** 2026-05-06

**Authors:** Thaynara de Lima Ribeiro, Aryane Ribeiro Oliveira, Bruna Mayara Faria Lima de Souza, Cristiane Maria Ascari Morgado, Luis Carlos Cunha Junior, André José de Campos

**Affiliations:** 1 Graduate Program in Agricultural Engineering, State University of Goiás (UEG), Anápolis 75132-903, Goiás, Brazil; 2 School of Agronomy, Federal University of Goiás (UFG), Goiânia 74690-900, Goiás, Brazil

**Keywords:** *Spondias purpurea*, postharvest technology, UV-C treatment, quality preservation, phenolic compounds, antioxidant activity

## Abstract

Seriguela (*Spondias purpurea* L.) is a tropical climacteric fruit characterized by high metabolic activity and rapid postharvest deterioration, which limits its shelf life and commercial distribution. Non-thermal technologies such as ultraviolet-C (UV-C) irradiation have been explored to delay ripening and improve the quality of fresh produce; however, information on the response of S. purpurea to UV-C treatment remains limited. This study evaluated the effects of different UV-C doses (0, 1, 2, 3, 4, and 5 kJ·m^−2^) on the postharvest quality and antioxidant properties of seriguela fruits stored at ambient temperature for eight days. Physical, physicochemical, and functional parameters were analyzed, including mass loss, firmness, color attributes (L*, chroma, and hue angle), soluble solids, pH, titratable acidity, vitamin C, total phenolics, and antioxidant activity (DPPH and ABTS). UV-C treatment significantly affected most quality attributes during storage. Intermediate doses (1–4 kJ·m^−2^) reduced mass loss, delayed softening, and improved color stability compared with untreated fruits. In addition, UV-C irradiation promoted the accumulation of bioactive compounds, with higher vitamin C levels at 1 kJ·m^−2^ and increased phenolic content and antioxidant activity at 2–4 kJ·m^−2^. Multivariate analysis confirmed that intermediate UV-C doses were associated with better preservation of overall fruit quality.

## 1. Introduction

The seriguela tree (*Spondias purpurea* L.) is a tropical fruit species belonging to the Anacardiaceae family, native to Central America and widely distributed in Mexico, the Caribbean, and countries in the northern region of South America. In Brazil, it occurs throughout most of the national territory, with greater predominance in the North and Northeast regions. The fruit is appreciated for its sweet flavor and characteristic aroma and is commonly cultivated in home orchards and marketed in regional markets, although it still lacks a structured commercial production chain [1]. The harvest season of seriguela occurs between December and February and, despite its regional economic relevance, its production remains predominantly extractive, resulting in limited technical information for postharvest management and difficulties in quality standardization [2]. During this period, the fruit generates significant economic activity, including the creation of informal jobs throughout the production chain, from harvesting to commercialization.

As a climacteric fruit, seriguela exhibits a high respiration rate and intense metabolic activity after harvest, which makes it highly perishable. When harvested at an immature physiological stage, the fruit does not properly develop its characteristic color and flavor, whereas when fully ripe it becomes highly susceptible to softening and rapid firmness loss, limiting its shelf life to only a few days [2,3]. Transport and storage conditions, combined with high temperatures, further aggravate postharvest losses, which may reach up to 50% of the total production [4,5].

In this context, the development of sustainable postharvest technologies is essential to extend the shelf life of seriguela and enable its insertion into broader markets. Conventional postharvest technologies, such as refrigeration, modified atmosphere storage, and edible coatings, have been widely used to extend fruit shelf life. However, these approaches may present limitations, including high operational costs, dependence on infrastructure, and potential alterations in sensory attributes. Ultraviolet-C (UV-C) radiation has been recognized as a promising non-thermal alternative for extending the shelf life of tropical fruits [6]. This technique, which uses short wavelengths (200–280 nm), acts through a physical and non-residual mechanism, reducing microorganisms on the fruit surface and stimulating beneficial physiological responses such as the accumulation of phenolic compounds and increased antioxidant activity [7,8]. Recent studies have also reported the effectiveness of UV-C treatments in the inactivation of surface fungi and bacteria in fruits and vegetables [9].

Based on these mechanisms, it is hypothesized that the application of intermediate UV-C doses (2–4 kJ·m^−2^) may modulate the metabolism of seriguela fruits, reducing deterioration and browning while maintaining their physicochemical and functional properties during storage. Similar effects have been reported in other fruits such as apricot, mango, avocado, and cajá-manga, in which UV-C treatments delayed ripening and contributed to the maintenance of external quality attributes, including color [10,11].

However, although the effects of UV-C irradiation have been extensively investigated in several fruit species, studies focusing on *Spondias purpurea* remain scarce, particularly under ambient storage conditions typical of tropical regions. Moreover, most available studies evaluate isolated quality parameters, with limited integration between physicochemical attributes, bioactive compounds, and antioxidant activity.

In this context, the present study advances current knowledge by providing a comprehensive evaluation of the postharvest responses of seriguela to different UV-C doses, integrating physical, physicochemical, and functional analyses, as well as multivariate approaches to better understand the metabolic behavior of the fruit during storage. Therefore, this study aimed to evaluate the effects of different UV-C doses on the postharvest quality, bioactive compounds, and antioxidant activity of seriguela fruits stored at ambient temperature.

## 2. Materials and Methods

### 2.1. Fruit Origin, Harvest, and Preparation

Seriguela fruits (*Spondias purpurea* L.) were harvested from a rural property located in the municipality of Caturaí, Goiás, Brazil (16°25′33″ S, 49°29′57″ W; 763 m altitude), approximately 83 km from Anápolis, Goiás, Brazil. Harvest. After harvesting, the fruits were placed in high-density polyethylene plastic containers and transported under refrigerated conditions (10 °C) for approximately 3 h from the harvest 960 fruits were collected. In the laboratory, the fruits were manually selected based on uniformity of color and size, and the absence of mechanical damage or symptoms of deterioration [12].

### 2.2. Experimental Design

Seriguela fruits were subjected to different doses of ultraviolet-C (UV-C) radiation, obtained by varying the exposure time to the irradiation source, and subsequently stored under controlled environmental conditions. The experiment was conducted in a completely randomized design in a 6 × 8 factorial scheme (six UV-C radiation doses × eight storage days), with four independent replicates per treatment, each replicate consisting of five fruits. The storage period was limited to seven days due to the high perishability of seriguela fruits under ambient conditions, which allows the evaluation of rapid postharvest changes and quality deterioration within a short time frame. The replicate was considered the experimental unit for statistical analysis, while individual fruits within each replicate were used for analytical determinations. For physicochemical analyses, fruits within each replicate were considered as subsamples. Non-destructive measurements were performed on individual fruits, while destructive analyses were carried out using composite samples obtained from fruits within each replicate. The number of fruits per replicate was defined based on standard practices in postharvest studies, balancing biological variability and analytical feasibility. The UV-C doses applied were 0 (control), 1, 2, 3, 4, and 5 kJ·m^−2^, determined according to different exposure times to the radiation source. The UV-C radiation source consisted of low-pressure mercury germicidal lamps emitting at approximately 254 nm. Radiation intensity was monitored using a Photo-Radiometer HD-2302-0 (Delta Ohm, Padua, Italy), positioned inside the irradiation chamber, allowing the calculation of the effective dose received by the fruits (W·m^−2^ converted to kJ·m^−2^). Exposure time was adjusted according to the measured radiation intensity to achieve the predefined UV-C doses. The selected UV-C dose range (0–5 kJ·m^−2^) was based on values commonly reported in the literature for postharvest treatment of fruits, particularly within levels considered effective for inducing beneficial responses without causing damage to plant tissues.

The UV-C irradiator consisted of a laboratory-built cylindrical chamber constructed of plastic polymer. The UV-C radiation source was provided by two 30 W germicidal lamps (without filter), emitting radiation at approximately 254 nm, installed at the upper and lower sections of the structure (0.5 × 0.5 × 0.9 m). The interior of the chamber was divided by a perforated galvanized mesh to ensure uniform exposure of the fruits. During UV-C treatment, fruits were arranged in a single layer without overlapping to minimize shading effects and ensure more uniform exposure of the fruit surface to radiation. The positioning of the lamps above and below the samples contributed to a more homogeneous distribution of UV-C light within the chamber. After treatment application, the fruits were stored under ambient conditions (26.8 ± 0.61 °C and 61.6 ± 3.88% relative humidity), which represent typical environmental conditions in tropical regions where seriguela is commonly handled and marketed without refrigeration. Similar storage conditions (~25 °C) have been used to evaluate postharvest behavior and rapid quality decline in highly perishable tropical fruits [12].

### 2.3. Physicochemical and Bioactive Analyses

#### 2.3.1. Mass Loss

Mass loss was determined by daily weighing using a precision digital balance (Shimadzu BL 3200H, Shimadzu Corp., Kyoto, Japan). The initial mass of each fruit (Mi) and the mass recorded on each evaluation day (Md) were used to calculate the percentage of mass loss according to Equation (1).

WL (%) = [(IM (g) − FM (g))/IM (g)] × 100(1)

WL = weight loss (%); IM (g) = initial mass; FM (g) = final mass.

#### 2.3.2. Fruit Firmness

Fruit firmness was determined by compression using a texture analyzer (Texture Analyzer CT3, Brookfield Engineering Laboratories, Middleboro, MA, USA) equipped with a cylindrical probe (5 mm diameter), operating at a speed of 7.0 mm·s^−1^ and a penetration depth of 5 mm. Measurements were performed on whole fruits without peeling in order to preserve the natural structure of the epidermis. Two measurements were performed per fruit, and the results were expressed in centinewtons (cN).

#### 2.3.3. Hydrogen Potential (pH)

The pH was determined using a portable potentiometer (Kasvi K39-0014P, range 0–14, São José doa Pinhais, Paraná, Brazil), calibrated with standard buffer solutions and equipped with automatic temperature compensation, according to the methodology described by AOAC [13].

#### 2.3.4. Titratable Acidity (TA)

Titratable acidity was determined by potentiometric titration using 5 g of homogenized pulp diluted in 100 mL of distilled water. The titration was performed with a standardized sodium hydroxide (NaOH) solution (0.1 mol·L^−1^) using 1% phenolphthalein as an indicator. The results were expressed as percentage of citric acid (% citric acid), according to AOAC methodology [13].

#### 2.3.5. Soluble Solids (SS)

The soluble solids content was determined by direct refractometry using a digital portable refractometer (Reichert Brix/RI-Chek, 0–62 °Brix, Depew, NY, USA). A few drops of seriguela juice were placed on the prism surface, and the results were expressed as °Brix according to AOAC methodology [13]. Soluble solids content was determined using juice obtained from a composite sample of five fruits per replicate.

#### 2.3.6. Ripening Index (SS/TA)

The ripening index was calculated as the ratio between soluble solids content (SS) and titratable acidity (TA), according to the methodology described by Tressler and Joslyn [14].

#### 2.3.7. Color Analysis

Fruit epidermis color was determined by diffuse reflectance using a portable colorimeter (Konica Minolta CR-400, Konica Minolta, Japan), calibrated with a standard white plate. Two readings were taken per fruit in the equatorial region. Measurements were expressed in the CIELAB color space, where L* represents luminosity (0 = black; 100 = white), a* represents the green (−a*) to red (+a*) axis, and b* represents the blue (−b*) to yellow (+b*) axis. Based on the a* and b* coordinates, chroma (C*) and hue angle (h°) were calculated according to Equations (2) and (3).

C* = (a*^2^ + b*^2^)^1/2^ (2)

h° = arctan (b*/a*)(3)

where a* represents the a* coordinate measured by the colorimeter, corresponding to the green (−a*) to red (+a*) axis, and b* represents the b* coordinate measured by the colorimeter, corresponding to the blue (−b*) to yellow (+b*) axis.

#### 2.3.8. Vitamin C (Ascorbic Acid)

The ascorbic acid content was determined according to the method described by Benassi and Antunes [15]. Briefly, 5 mL of seriguela juice were transferred to a 50 mL volumetric flask and diluted with a 0.5% (*w*/*v*) oxalic acid extraction solution. After filtration of the pulp through gauze, a 10 mL aliquot was titrated with a 0.02% 2,6-dichlorophenolindophenol solution until the appearance of a light pink coloration, indicating the endpoint of the titration. The results were expressed as mg of ascorbic acid per 100 g of fresh pulp (mg ascorbic acid 100 g^−1^ fresh weight).

#### 2.3.9. Total Phenolic Compounds and Antioxidant Activity (DPPH and ABTS)

Extracts were prepared according to a method adapted from Larrauri et al. [16]. Briefly, 2.5 g of seriguela pulp were weighed and mixed with 10 mL of 50% methanol (*v*/*v*). The mixture was homogenized and kept at room temperature for 60 min. Subsequently, the solution was centrifuged (K14-0815, Kasvi, Brazil) at approximately 1790× *g* for 30 min, and the supernatant was transferred to a 25 mL volumetric flask. The residue was subjected to a second extraction with 10 mL of 70% acetone (*v*/*v*), repeating the same homogenization, resting, and centrifugation steps. The supernatants were combined and the final volume was adjusted with distilled water.

Total antioxidant activity was determined using the free radical scavenging methods DPPH (2,2-diphenyl-1-picrylhydrazyl) and ABTS (2,2′-azinobis (3-ethylbenzothiazoline-6-sulfonic acid) diammonium salt), according to Rufino et al. [17]. For the DPPH assay, absorbance was measured at 515 nm after 11 min of reaction using methanol as the blank. Results were expressed as EC_50_ (g sample g^−1^ DPPH), representing the mass of sample required to reduce 50% of the free radical. For the ABTS assay, absorbance was measured at 734 nm after 6 min of reaction using ethanol as the blank. Results were expressed as µmol Trolox g^−1^ fresh weight (µmol Trolox g^−1^ FW).

#### 2.3.10. Total Extractable Polyphenols (TEPs)

Extracts were obtained according to the procedure previously described for total antioxidant activity determination. The content of total extractable polyphenols (TEPs) was determined by UV–Vis spectrophotometry using the Folin–Ciocalteu reagent, according to Obanda et al. [18], with adaptations described by Rufino et al. [17]. A calibration curve was prepared using standard solutions of gallic acid (GAE, gallic acid equivalents) at concentrations ranging from 10 to 50 µmol·L^−1^. For each sample, 0.1 mL of extract was mixed with 1 mL of Folin–Ciocalteu reagent, 2 mL of 20% sodium carbonate solution (*w*/*v*), and 2.9 mL of distilled water. The mixture was allowed to react for 30 min at room temperature, and absorbance was measured using a UV–Vis spectrophotometer (SP-1105, Tecnal, Piracicaba, Brazil) at 700 nm. For the blank, 1 mL of distilled water was used instead of the extract. Results were expressed as mg of gallic acid equivalents per 100 g of fresh pulp (mg GAE 100 g^−1^ FW).

### 2.4. Statistical Analysis

Results were expressed as mean ± standard deviation (SD). Data were subjected to analysis of variance (ANOVA), and when significant effects were detected (*p* < 0.05). When applicable, polynomial regression models were fitted to describe the behavior of variables during storage. Univariate analyses (ANOVA and regression) were performed using SISVAR software (version 5.6). Pearson correlation coefficients (r) and multivariate analyses were conducted in Python (Version 3.10). Principal component analysis (PCA) was performed using the scikit-learn library (Version 1.3.0; sklearn.decomposition.PCA) after data standardization with StandardScaler. Hierarchical cluster analysis (HCA) was carried out using scipy.cluster.hierarchy (Version 1.11.0) with Euclidean distance and Ward’s linkage method. A correlation heatmap was generated using the seaborn (version 0.12.2) and matplotlib (version 3.7.1) libraries. Additional statistical calculations were performed using pandas (version 2.0.3), numpy (version 1.24.3), and scipy.stats (version 1.11.0; pearsonr) (pearsonr).

## 3. Results and Discussion

### 3.1. Mass Loss

**Figure 1:**
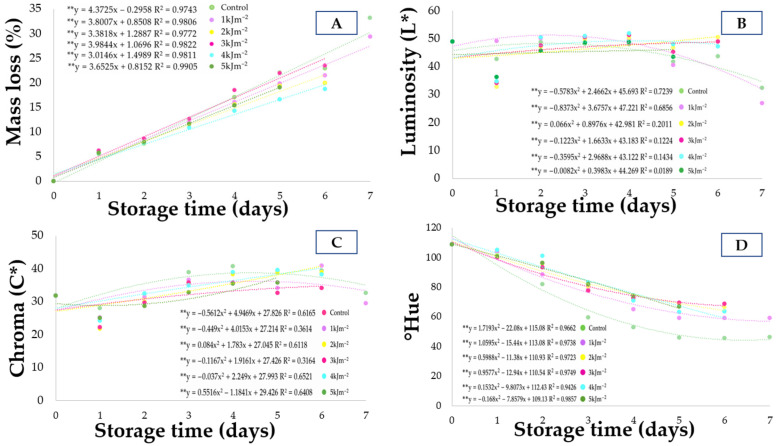
Temporal evolution of postharvest quality attributes of seriguela (*Spondias purpurea* L.) subjected to different UV-C doses during ambient storage. (**A**) Mass loss (%); (**B**) Luminosity (L*); (**C**) Chroma; and (**D**) Hue angle (°hue) of fruits treated with 0, 1, 2, 3, 4, and 5 kJ·m^−2^ of UV-C and stored for 7 days at 26.8 ± 0.61 °C and 61.6 ± 3.88% relative humidity. Dotted lines represent the fitted polynomial regression models for each treatment. Data are expressed as mean values of four replicates. “**” indicates statistical significance at *p* ≤ 0.01.

**Figure 2:**
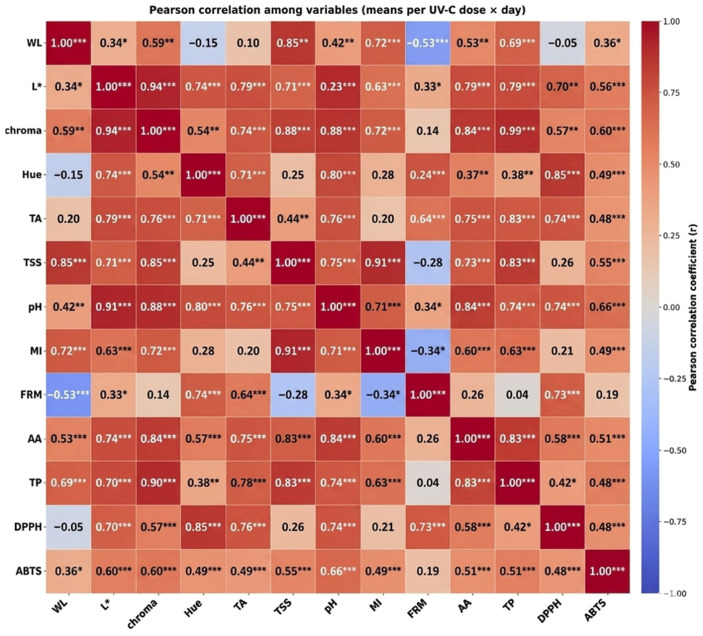
Pearson correlation matrix among physicochemical and bioactive variables of seriguela (*Spondias purpurea* L.) subjected to different UV-C doses across storage days. Heatmap showing the Pearson correlation coefficients (r) among all evaluated variables: mass loss (WL), luminosity (L*), chroma, hue angle (Hue), titratable acidity (TA), soluble solids (TSS), pH, ripening index (MI), firmness (FRM), ascorbic acid (AA), total phenolics (TP), antioxidant activity by DPPH, and antioxidant activity by ABTS. Color scale ranges from –1 (strong negative correlation) to +1 (strong positive correlation). Significance levels are indicated as *p* < 0.05 (*), *p* < 0.01 (**), and *p* < 0.001 (***).

As shown in Figure 1A, mass loss increased linearly over the storage period for all treatments. Regression models fitted to the data confirmed the progressive increase in water loss during storage, reflecting the typical dehydration pattern of climacteric fruits. The effects of UV-C dose, storage time, and their interaction were evaluated by analysis of variance (ANOVA), which indicated that [UV-C dose had no significant effect/had a significant effect on mass loss (*p* < 0.05)]. Differences in the slopes of the regression curves indicate that UV-C doses influenced the rate of mass loss throughout storage. The lowest slopes of the regression curves were observed for the doses of 2 and 4 kJ·m^−2^, indicating a lower rate of mass loss during storage compared with the control treatment, whose regression equation presented the highest slope, indicating the greatest mass loss over time. This behavior suggests that exposure to intermediate doses of UV-C radiation contributed to maintaining epidermal integrity and the cuticular barrier, thereby reducing transpiration and water loss. Similar results were reported by Sanches et al. [19], who associated this effect with changes in respiratory metabolism and reduced degradation of pectins in the cell wall. The negative correlation observed between mass loss and firmness (Figure 2) indicates that tissue dehydration is associated with loss of turgor and fruit softening. Conversely, the positive correlations with soluble solids (SS) (r = 0.85) and luminosity (L*) (r = 0.34) suggest that the progression of water loss is accompanied by an increase in soluble solids concentration and peel lightness, which are typical characteristics of the ripening process. Similar effects have been reported in other fruits treated with UV-C radiation. For example, UV-C treatments have been shown to delay quality deterioration and maintain postharvest attributes in fruits such as mango and pineapple during storage [11,20]. These findings support the potential of UV-C as a non-thermal technology to slow physiological changes and extend the shelf life of fresh fruits.

Therefore, the lower mass loss observed in fruits treated with 1 and 4 kJ·m^−2^ suggests delayed ripening and greater preservation of fruit structural integrity. Similar results were reported by Ramos et al. [21], who observed a progressive increase in mass loss during storage when evaluating the effect of ultraviolet radiation on the postharvest quality of lychee fruits. It is important to note that the increase in mass loss during storage may lead to a concentration effect when results are expressed on a fresh weight basis, which should be considered when interpreting changes in bioactive compounds.

### 3.2. Color

The polynomial regression models fitted to luminosity values indicate a gradual increase during the initial storage days followed by stabilization or decline depending on the UV-C dose, suggesting that radiation influenced pigment degradation dynamics during ripening (Figure 1B–D). This behavior may be associated with the modulation of oxidative metabolism induced by UV-C radiation, which can delay senescence-related pigment degradation processes. Visual appearance is one of the main quality attributes perceived by consumers, and luminosity (L*) expresses the intensity of surface color, ranging from 0 (black) to 100 (white), thus serving as an indicator of freshness and epidermal integrity [22]. As illustrated in Figure 1B, fruits subjected to intermediate UV-C radiation doses (2, 3, and 4 kJ·m^−2^) maintained relatively stable luminosity values throughout storage, with a slight increase until the sixth day. This behavior indicates a delay in the natural darkening of the peel, suggesting reduced pigment degradation and preservation of fruit surface integrity. This response is consistent with a hormetic effect of UV-C radiation, in which moderate doses induce protective physiological responses without causing cellular damage. According to Pott et al. [23], cuticular darkening is a physiological process associated with ripening, resulting from chlorophyll degradation and phenolic oxidation. Therefore, the stability observed at these UV-C doses may be related to the modulatory effect of radiation on oxidative enzymes such as polyphenol oxidase and peroxidase, thereby reducing surface browning. In addition, the maintenance of luminosity may be linked to the preservation of membrane integrity, which limits substrate–enzyme interactions involved in enzymatic browning. In contrast, the control treatment and the 1 kJ·m^−2^ dose showed an initial increase in L until the second day, followed by a marked decrease until the end of storage, indicating loss of brightness and progression of ripening. This decline is consistent with physiological processes commonly observed in climacteric fruits, such as increased ethylene production and respiratory activity, which are associated with chlorophyll degradation and the development of yellowish and reddish tones [19]. Similar results were reported by Cabia et al. [24] in ‘Hass’ avocados and by Dias et al. [25] in ‘Gala’ apples, who observed a decreasing trend in luminosity during storage after UV-C treatment. Pearson correlation analysis (Figure 2) showed that luminosity (L*) exhibited a strong and highly significant positive correlation with chroma and hue angle, indicating that increases in L* are directly associated with greater color intensity and uniformity. This relationship reinforces that color evolution is a coordinated process involving pigment transformation and optical properties of the fruit surface during ripening. Additionally, L* showed positive correlations with total phenolics and antioxidant activity, suggesting that treatments capable of preserving fruit coloration also contributed to maintaining bioactive compounds and antioxidant capacity. These findings suggest that UV-C radiation not only delays visual deterioration but also contributes to maintaining the functional quality of the fruits through the activation of antioxidant defense mechanisms.

The hue angle (°Hue) showed a marked decreasing trend throughout storage for all treatments (Figure 1D), indicating a progressive shift in peel color from yellowish to orange-red tones, which is characteristic of the ripening process in seriguela fruits. According to Schiavon et al. [22], hue angle is an important parameter for describing color changes during fruit ripening, as it reflects shifts in the predominant wavelength perceived in the fruit surface. The more pronounced reduction observed in the control treatment suggests a faster progression of ripening, likely associated with increased chlorophyll degradation and carotenoid synthesis. In contrast, fruits treated with intermediate UV-C doses (2–4 kJ·m^−2^) showed a slower decline in °Hue, indicating a delay in pigment transformation and suggesting a modulatory effect of UV-C radiation on ripening-related metabolic processes. This behavior reinforces the role of UV-C treatment in preserving visual quality and delaying senescence in seriguela fruits.

Chroma represents the saturation or purity of color and is directly associated with visual intensity and fruit attractiveness, with higher values indicating more vivid colors typically observed in ripe fruits [22]. As shown in Figure 1C, chroma values increased progressively in all treatments until the fifth day of storage, followed by a slight decrease at the end of the storage period. This behavior indicates the gradual transition from green coloration to orange tones, which are characteristic of seriguela ripening. Control fruits presented the highest chroma values on the last day of storage (31.71–32.56), indicating advanced ripening. In contrast, intermediate UV-C radiation doses (2–4 kJ·m^−2^) delayed this increase, suggesting a protective effect against premature chlorophyll degradation and accelerated carotenoid synthesis. Similar results were reported by McCook-Russell et al. [26], who observed a significant increase in chroma values in tropical fruits during ripening stages, indicating greater color purity and saturation as physiological ripening progressed.

Pearson correlation analysis (Figure 2) showed that chroma exhibited a highly significant positive correlation with L* and a moderate correlation with hue angle, indicating that color intensification is associated with increased luminosity and changes in hue angle during ripening. Furthermore, chroma showed a strong positive correlation with total phenolics and antioxidant activity determined by the ABTS assay, suggesting that the preservation of fruit coloration is associated with the maintenance of phenolic compounds and antioxidant capacity. These relationships indicate that intermediate doses of UV-C radiation positively influenced color stability and antioxidant mechanisms, contributing to the maintenance of both visual and functional quality of seriguela fruits during storage.

### 3.3. Firmness

**Figure 3:**
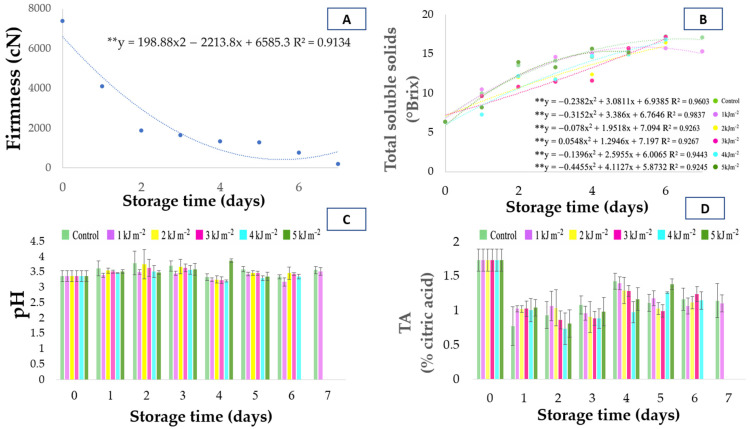
Temporal evolution of physicochemical attributes of *Spondias purpurea* fruits subjected to different UV-C doses during storage at ambient temperature. (**A**) Firmness (cN); (**B**) Soluble solids content (°Brix); (**C**) TA (Titratable acidity, % citric acid); and (**D**) pH. Each point or bar represents the mean ± standard deviation (*n* = 4). Polynomial regression models (*p* < 0.05) are shown for variables with significant time effects. “**” indicates statistical significance at *p* ≤ 0.01.

Regarding fruit firmness (Figure 3A), no significant interaction was observed between the evaluated factors, with an isolated effect detected only for storage days. A pronounced reduction in firmness was observed throughout the storage period, decreasing from 7369.69 to 188.04 cN, which indicates the progression of ripening and the loss of tissue resistance. This marked decline reflects the transition from physiological maturity to senescence, during which structural integrity of the fruit is progressively compromised. This behavior is consistent with the trend observed for mass loss (Figure 1A), suggesting that firmness reduction is associated with loss of cellular turgor and tissue dehydration resulting from water loss during storage [27]. This reduction in firmness is commonly associated with cell wall degradation mediated by pectinolytic enzymes during fruit ripening. In addition, water loss may intensify cell wall disassembly by promoting tissue shrinkage and increasing mechanical stress within the fruit structure.

According to Pearson correlation analysis (Figure 2), firmness showed a significant negative correlation with mass loss and a positive correlation with pH, reinforcing the relationship between water loss, ripening progression, and cellular disintegration. Similar results were reported by Martins et al. [28], who observed a marked reduction in firmness in seriguela fruits stored under refrigeration for five days, associated with fruit shriveling and peel wrinkling. Likewise, Pan and Zu [29], when evaluating the effect of UV-C radiation on minimally processed pineapple, reported a progressive decrease in firmness during storage regardless of the applied dose. The authors attributed this reduction to gradual water loss and structural weakening of the tissues, which is consistent with the behavior observed in seriguela fruits in the present study. However, the absence of a significant UV-C effect on firmness in this study suggests that the applied doses were not sufficient to markedly inhibit the activity of cell wall-degrading enzymes or to significantly alter the softening kinetics of the fruit. This pattern of firmness decline can be explained by the physiological processes associated with fruit ripening. Cell wall degradation is one of the main mechanisms involved in fruit softening, as it involves the hydrolysis of pectins and hemicelluloses mediated by enzymes such as polygalacturonase, pectin methylesterase, and cellulase. As these enzymes reduce cell-to-cell adhesion, tissue structure becomes less resistant, intensifying fruit softening. These enzymatic processes are typically regulated by ethylene in climacteric fruits, which accelerates softening during the later stages of storage. Thus, the loss of firmness observed in seriguela fruits reflects both the natural progression of ripening and the reduction in cellular turgor, phenomena widely reported for climacteric tropical fruits during postharvest storage [30]. From a practical perspective, this rapid firmness loss indicates a limited shelf life under ambient conditions, highlighting the importance of combining UV-C treatment with other preservation strategies to effectively delay textural degradation.

### 3.4. Soluble Solids

The soluble solids content (TSS) increased continuously during storage for all UV-C radiation doses and for the control treatment (Figure 3B). Polynomial regression analysis also confirmed this increasing trend, indicating a progressive accumulation of soluble sugars during storage. Differences in regression patterns among treatments suggest that UV-C exposure modulated the rate of sugar accumulation during fruit ripening. This modulation indicates that UV-C radiation may influence carbohydrate metabolism, potentially altering the balance between sugar synthesis, degradation, and utilization during storage.

At the beginning of the storage period, fruits presented average values of 6.32 °Brix, reaching 17.1 °Brix in the control and 15.2 °Brix at the dose of 1 kJ·m^−2^ at the end of the experiment. This increase indicates the natural ripening process of the fruits, associated with the conversion of starch and polysaccharides into reducing sugars, as well as with the concentration of solutes resulting from water loss during storage [31]. In addition, the increase in TSS may be partially enhanced by the concentration effect caused by moisture loss, which should be considered when interpreting compositional changes during storage. In general, fruits treated with intermediate doses (2, 3, and 4 kJ·m^−2^) showed slightly lower TSS values until the sixth day of storage, suggesting a delay in the ripening process caused by UV-C exposure. This behavior is consistent with a delay in ripening-related metabolic processes, as commonly reported for climacteric fruits, resulting in lower degradation of carbohydrate reserves and a slower accumulation of soluble sugars during storage. This response may reflect a reduction in metabolic activity and ethylene production, resulting in slower conversion of complex carbohydrates into soluble sugars.

Pearson correlation analysis (Figure 2) revealed a significant positive correlation between TSS and pH and between TSS and the ripening index (MI), indicating that increases in soluble solids accompany the progression of ripening and the reduction in acidity. A weak negative correlation with firmness was also observed, suggesting that sugar accumulation tends to occur simultaneously with pulp softening. This relationship highlights the coordinated nature of ripening, in which biochemical changes (sugar accumulation) occur simultaneously with structural changes (tissue softening). These results corroborate the typical behavior of climacteric fruits, in which a progressive increase in soluble solids occurs due to the conversion of reserve carbohydrates into simple sugars during ripening [32]. From a practical perspective, the slower increase in TSS observed at intermediate UV-C doses suggests a potential delay in the development of sweetness, which may be associated with extended shelf life and slower ripening kinetics.

### 3.5. pH

In general, pH values remained within the range of 3.3 to 3.6 throughout the storage period (Figure 3C), indicating maintenance of the natural acidity characteristic of the species. Recent studies conducted with different ecotypes of *Spondias purpurea* have also reported similar pH ranges, varying according to ripening stage and genetic material. The absence of significant variation in pH under UV-C treatments suggests that the radiation did not substantially affect the activity of enzymes involved in the utilization of respiratory organic acids or their degradation rate. Therefore, the reduction in acidity was mainly determined by the basal respiration of the fruit and by typical biochemical changes associated with the ripening process.

### 3.6. Titratable Acidity

Titratable acidity (TA) is one of the main parameters used to express the content of organic acids in fruits and is directly related to ripening stage and sensory perception of flavor. As shown in Figure 3D, TA decreased in all treatments during storage, with initial average values of 1.73 ± 0.16% citric acid, decreasing to 1.14 ± 0.24% in the control and 1.10 ± 0.12% at the dose of 1 kJ·m^−2^ at the end of the storage period. This reduction is typical of climacteric fruits and reflects the consumption of organic acids as substrates in respiratory metabolism and sugar synthesis during ripening [33,34]. The fact that UV-C doses did not significantly alter the rate of TA decline suggests that the treatment did not markedly affect the overall metabolic processes associated with organic acid utilization during ripening, as commonly reported for climacteric fruits [35]. This metabolic stability indicates that UV-C treatments at the applied doses prevented cellular disorganization and accelerated senescence, preserving the normal ripening kinetics of the fruits. Pearson correlation analysis (Figure 2) revealed a significant positive correlation between pH and TSS and between pH and the ripening index (MI), indicating that increases in pH accompany increases in soluble solids and ripening index, parameters associated with ripening progression and sweeter flavor. A weak positive correlation between pH and firmness was also observed, suggesting that variations in pH may occur simultaneously with structural changes in fruit tissues during storage. The gradual reduction in firmness combined with the increase in pH indicates the physiological progression of fruit ripening.

The inverse relationship between titratable acidity and pH observed in this study reflects the expected physiological behavior: as organic acids are metabolized during ripening, pH increases while titratable acidity decreases. This pattern was also confirmed by Pearson correlation analysis (Figure 2), in which TA showed a strong negative correlation with pH and a positive correlation with firmness, indicating that firmer fruits tend to retain higher levels of organic acids.

### 3.7. Ripening Index (TSS/TA)

**Figure 4:**
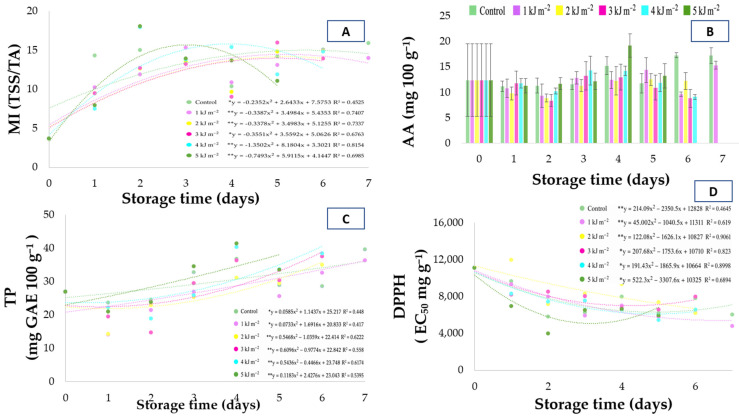
(**A**) Maturation index (TSS/TA) of *Spondias purpurea* fruits subjected to different UV-C doses during storage, (**B**) AA (Ascorbic acid vitamin C) content (mg 100 mL^−1^) as a function of UV-C dose and storage time, (**C**) TP (Total extractable phenolics; mg GAE 100 g^−1^) in fruits exposed to UV-C irradiation throughout storage, (**D**) Antioxidant activity determined by the DPPH method (EC_50_ mg g^−1^ DPPH) across UV-C doses and storage days. Polynomial regressions are shown for each UV-C treatment when significant (*p* < 0.05). “*” indicates statistical significance at *p* ≤ 0.05; “**” indicates statistical significance at *p* ≤ 0.01.

The ripening index (MI), defined as the ratio between soluble solids and titratable acidity (TA), is widely recognized as one of the main indicators of fruit ripening stage, as it simultaneously integrates the increase in sugars and the reduction in organic acids during postharvest storage. For seriguela fruits, a gradual increase in MI was observed throughout the storage period (Figure 4A). Until approximately the fifth day of storage, fruits from the control treatment and those treated with 4 kJ·m^−2^ presented the highest MI values, reflecting the combination of higher soluble solids accumulation and a more pronounced reduction in acidity, which indicates a faster progression of ripening. In contrast, fruits treated with 1 kJ·m^−2^ maintained lower MI values throughout storage, suggesting a moderate delay in the ripening process. The treatment with 5 kJ·m^−2^ showed a distinct behavior, with an early increase in MI around the third day and termination of storage on the fifth day, indicating possible acceleration of ripening and reduction in shelf life, likely associated with physiological stress caused by the higher UV-C radiation intensity. These trends are supported by Pearson correlation analysis (Figure 2), in which the ripening index showed a very strong positive correlation with soluble solids, demonstrating that increases in MI accompany the accumulation of soluble sugars during ripening. In contrast, the correlation between MI and titratable acidity was weak, suggesting that, in the present study, variations in the ripening index were more strongly influenced by soluble solids behavior than by changes in fruit acidity. This relationship indicates that although TA showed a decreasing trend during storage, the rate of sugar accumulation exerted a greater influence on MI variation. Thus, the reduction in acidity accompanies the progression of ripening, tissue softening, and the progressive accumulation of sugars. In terms of sensory quality, the reduction in TA, together with the increase in soluble solids (as discussed previously), resulted in an increase in the TSS/TA ratio, which is considered one of the most representative indicators of fruit flavor development [14]. The gradual increase in this ratio under UV-C treatments confirms that irradiation preserved the natural evolution of the flavor profile of seriguela fruits, promoting fruits that are less acidic and more palatable throughout the storage period.

Principal component analysis (PCA) revealed that the ripening index (MI) clustered with variables associated with ripening progression, such as soluble solids (TSS), total phenolic compounds (TP), and color parameters (chroma and hue angle). In contrast, it was positioned opposite to variables related to initial fruit freshness, such as firmness and mass loss. This pattern indicates that the ripening index acts as a robust marker of metabolic changes associated with fruit ripening and highlights the ability of multivariate analysis to discriminate different physiological stages of the fruits during storage.

### 3.8. Vitamin C (Ascorbic Acid), Total Phenolic Compounds (TP), and Antioxidant Activity (DPPH)

Vitamin C contents showed fluctuations during storage for all evaluated treatments (Figure 4B). The polynomial regression curves observed in Figure 4 also demonstrate these fluctuations over storage time, indicating dynamic changes in antioxidant metabolism during fruit ripening and stress responses induced by UV-C exposure. These fluctuations suggest a balance between biosynthesis and degradation processes, which are strongly influenced by the oxidative status of the fruit tissues during storage.

As observed, the ascorbic acid content tended to increase during the initial stages of storage, reaching a peak around Day 4, and subsequently decreased. This behavior may be associated with the biosynthesis of ascorbic acid from metabolic precursors such as D-glucose and D-galactose during respiratory metabolism. Such variation is characteristic of ripening fruits, as ascorbic acid acts as a primary antioxidant involved in the neutralization of reactive oxygen species generated during oxidative metabolism [34,36]. The initial increase may be interpreted as an adaptive response to oxidative stress, in which the fruit enhances its antioxidant capacity to maintain cellular redox balance. Such variation is characteristic of ripening fruits, as ascorbic acid acts as a primary antioxidant involved in the neutralization of reactive oxygen species generated during oxidative metabolism. Although the UV-C radiation doses did not promote a statistically significant increase in vitamin C content, suggesting that the radiation intensity or exposure time was not sufficient to trigger a strong stress-induced antioxidant response, it is noteworthy that the contents at higher doses (3, 4, and 5 kJ·m^−2^) remained comparable to or even higher than those observed in the control at certain storage times. This behavior may indicate a mild stress effect induced by UV-C radiation, which was sufficient to maintain antioxidant levels without causing oxidative damage to cellular structures. The absence of significant degradation, even under abiotic stress induced by UV-C radiation, suggests that the tested doses are safe from a nutritional standpoint. This reinforces the potential application of UV-C as a postharvest technology capable of preserving nutritional quality while modulating fruit metabolism.

Pearson correlation analysis (Figure 2) showed a positive association between ascorbic acid and pH, suggesting that the increase in vitamin C accompanies ripening progression and the reduction in fruit acidity. A strong positive correlation was also observed between ascorbic acid and total phenolics, as well as a moderate correlation with antioxidant activity determined by the ABTS assay. These results indicate that ascorbic acid contributes to the overall antioxidant capacity of the fruit, acting together with phenolic compounds to delay oxidative processes associated with ripening and senescence. This synergistic interaction between antioxidant compounds highlights a coordinated defense mechanism against oxidative stress, which is essential for maintaining fruit quality during postharvest storage.

Total phenolic compounds (Figure 4C) showed an increasing trend during storage for all treatments. Seriguela fruits exhibited initial average values of 26.92 mg 100 mL^−1^, reaching 39.64 mg 100 mL^−1^ in the control and 36.37 mg 100 mL^−1^ at the dose of 1 kJ·m^−2^ at the end of the storage period. Among the UV-C treatments, the dose of 4 kJ·m^−2^ showed elevated phenolic compound levels around the fifth day of storage, indicating a possible metabolic stimulation induced by radiation. This increase suggests that ripening associated with UV-C exposure may be associated with increased accumulation of total phenolic compounds. This response may be related to a hormetic effect, in which moderate UV-C doses act as a controlled abiotic stress, potentially triggering defense-related metabolic responses without causing cellular damage.

Phenolic compounds are among the most active antioxidants in plant tissues, and their increase contributes to preserving antioxidant capacity by reducing the impact of free radicals and delaying senescence [37]. The accumulation of phenolics during storage may be associated with metabolic responses commonly reported in the literature, rather than specific enzymatic pathways evaluated in this study. The accumulation of phenolics during storage may also be associated with the activation of key enzymes such as phenylalanine ammonia-lyase (PAL), which plays a central role in the phenylpropanoid pathway. Pearson correlation analysis (Figure 2) showed significant positive correlations between total phenolics (TP) and antioxidant activity. A strong positive correlation was also observed between TP and vitamin C, suggesting a synergistic interaction between these compounds in maintaining the antioxidant capacity of the fruits. This coordinated increase in antioxidant compounds indicates an integrated defense system against oxidative stress, in which different classes of metabolites act complementarily to maintain cellular redox homeostasis. These results confirm that the increase in total phenolic content is directly associated with the enhancement of total antioxidant capacity, indicating a synergistic role of phenolics and ascorbic acid in cellular protection and in maintaining fruit quality. The increase in total phenolic compounds in seriguela fruits suggests that intermediate UV-C radiation doses (especially 4 kJ·m^−2^) may be associated with enhanced antioxidant responses, contributing to the maintenance of fruit quality and functional potential during storage. In contrast, lower or higher doses may be less effective, either due to insufficient stimulus or potential metabolic imbalance, reinforcing the importance of dose optimization in UV-C applications. Similar results were reported for ‘Napoleon’ grapes treated with UV-C, which showed a significant increase in phenolic compounds during storage [37]. Erkan et al. [38] observed a comparable response in strawberries, indicating that moderate UV-C doses may be associated with increased accumulation of phenolic compounds. From a practical standpoint, these findings highlight the potential of UV-C treatment as a non-chemical strategy to enhance the functional quality of fruits by increasing bioactive compounds during postharvest storage.

The evolution of antioxidant activity determined by the DPPH method (Figure 4D) was expressed as EC_50_, a parameter that represents the concentration of extract required to neutralize 50% of the free radicals present in the reaction system. In this context, lower EC_50_ values indicate higher antioxidant capacity, since a smaller amount of sample is required to promote DPPH radical scavenging. During storage, variations in EC_50_ values were observed among treatments, reflecting changes in the antioxidant capacity of the fruits throughout the ripening process. These variations indicate that antioxidant capacity is dynamically regulated during storage, being closely associated with metabolic adjustments in response to oxidative stress. Until approximately the fourth day of storage, fruits treated with 5 kJ·m^−2^ presented the lowest EC_50_ values, indicating greater antioxidant capacity during this initial period. Subsequently, the dose of 1 kJ·m^−2^ exhibited the lowest EC_50_ values until the end of the experiment, suggesting a more prolonged maintenance of antioxidant activity in these fruits. This shift in antioxidant response among doses suggests that higher UV-C doses may induce an early but transient stimulation, whereas lower doses promote a more sustained metabolic response over time. This behavior may be associated with the effect of UV-C radiation as a moderate oxidative stress stimulus, which may promote antioxidant responses and the accumulation of secondary metabolites, such as phenolic compounds. This pattern is consistent with a hormetic response, in which controlled abiotic stress enhances the antioxidant system without causing oxidative damage to the tissues. This interpretation is supported by Pearson correlation analysis (Figure 2), which revealed a positive correlation between antioxidant activity determined by DPPH and total phenolics, as well as a positive correlation with vitamin C, indicating that the antioxidant capacity of the fruits is associated with the accumulation of these bioactive compounds during storage. These results reinforce the integrated role of different antioxidant systems, in which phenolic compounds and ascorbic acid act synergistically to scavenge reactive oxygen species and maintain cellular redox balance. Furthermore, the increase in antioxidant capacity observed during storage suggests that postharvest conditions, combined with UV-C treatment, may enhance the functional quality of seriguela fruits.

On the last day of storage, the dose of 1 kJ·m^−2^ presented the lowest EC_50_ value (4784.86 mg g^−1^ DPPH), corresponding to the highest antioxidant efficiency, whereas the control exhibited a higher EC_50_ value (6017.09 mg g^−1^ DPPH), confirming the beneficial effect of low-dose UV-C radiation on the preservation of antioxidant activity. These results are consistent with observations reported for other fruits of the Anacardiaceae family, such as umbu, cajá, and cashew, whose antioxidant activities range from 7.074 to 9.397 EC_50_ mg g^−1^ DPPH [17]. This similarity reinforces the natural antioxidant potential of seriguela and suggests that UV-C treatment may help maintain this property during postharvest storage by delaying the degradation of compounds responsible for antioxidant activity. Overall, the behavior of antioxidant activity throughout storage indicates that moderate UV-C doses exert a protective role and induce biochemical defense responses, contributing to the maintenance of antioxidant functionality in seriguela fruits without compromising their sensory quality.

**Figure 5:**
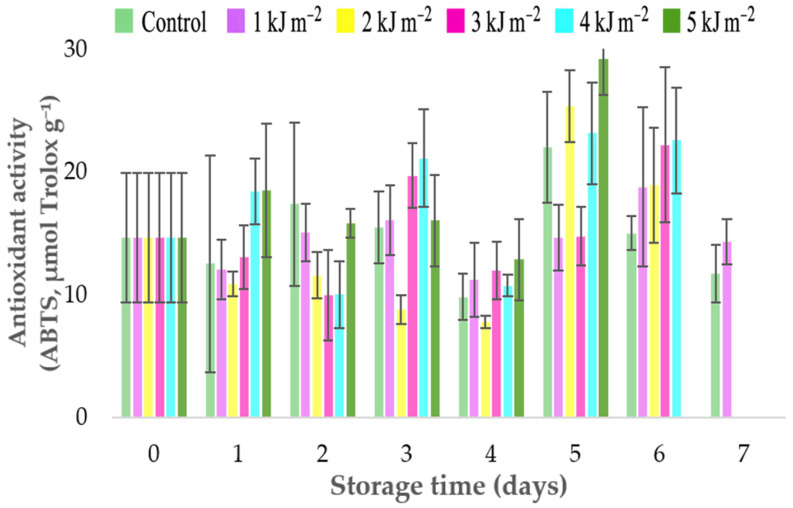
Antioxidant activity (ABTS assay, µmol Trolox g^−1^) in *Spondias purpurea* L. fruits exposed to different UV-C doses (0, 1, 2, 3, 4, and 5 kJ·m^−2^) during 7 days of storage at room temperature. Values represent mean ± standard deviation.

The antioxidant activity determined by the ABTS method (Figure 5) showed variations throughout the storage period, without a clearly defined linear trend among treatments. Fluctuations in antioxidant capacity were observed over time, with some treatments showing temporary increases followed by reductions on subsequent days. Higher values were observed on the fifth day of storage, particularly in fruits treated with 5 kJ × m^−2^, indicating a temporary increase in antioxidant capacity under this condition. In the final days of storage, some treatments, such as 1 kJ·m^−2^, maintained relatively higher antioxidant activity values compared with the control, suggesting that moderate UV-C doses may contribute to maintaining the antioxidant capacity of the fruits. This variation may be associated with the behavior of total phenolic compounds, which also showed an increasing trend during storage (Figure 4C). These metabolites are considered important contributors to antioxidant activity in tropical fruits [30]. Indeed, Pearson correlation analysis (Figure 2) revealed a positive correlation between antioxidant activity measured by ABTS and total phenolics, as well as a positive correlation with vitamin C, indicating that antioxidant capacity is associated with the accumulation of these bioactive compounds during storage. The interaction between phenolic compounds and ascorbic acid may explain the fluctuations observed in antioxidant activity during ripening, reflecting the balance between the synthesis of antioxidant metabolites and their utilization in defense processes against oxidative stress.

Similar responses have been reported in other fruit species subjected to UV-C irradiation. In mango, moderate UV-C doses between approximately 2 and 5 kJ·m^−2^ reduced weight loss, maintained firmness, and promoted antioxidant responses during storage, demonstrating the effectiveness of intermediate doses in preserving fruit quality [39]. Likewise, studies on fruits and vegetables have shown that UV-C exposure can stimulate the accumulation of phenolic compounds and enhance antioxidant activity by activating defense-related metabolic pathways [9]. These responses are generally attributed to a hormetic effect of UV-C radiation, in which moderate doses induce controlled oxidative stress that stimulates the phenylpropanoid pathway and the synthesis of antioxidant metabolites [36]. Therefore, the increase in phenolic compounds and antioxidant activity observed in seriguela fruits appears consistent with physiological responses reported for other fruits treated with moderate UV-C doses.

Although UV-C treatment showed beneficial effects on quality preservation, it is important to consider that excessive exposure may lead to adverse effects, such as superficial tissue damage and changes in sensory attributes, including the development of bitterness associated with the accumulation of phenolic compounds. However, no visible symptoms of damage were observed in the present study at the evaluated doses, suggesting that the applied UV-C levels were within a safe range for maintaining fruit quality.

### 3.9. Principal Component Analysis and Hierarchical Clustering

**Figure 6:**
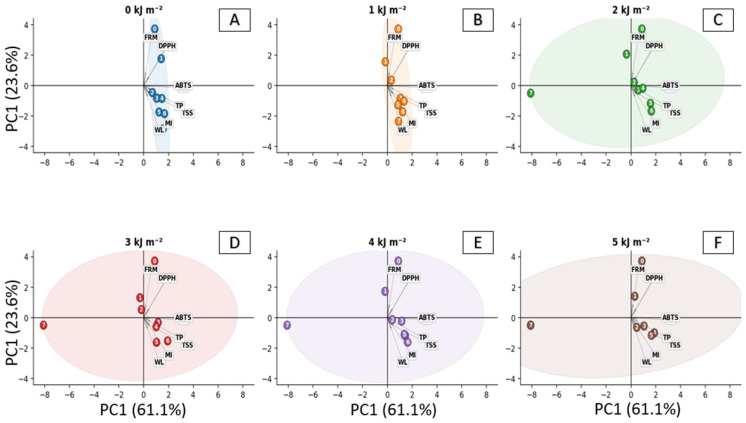
Principal component analysis (PCA) of physicochemical and functional variables of seriguela fruits subjected to UV-C radiation. (**A**) General biplot showing the distribution of samples according to the first two principal components (PC1 = 61.1%; PC2 = 23.6%), with vectors representing the original variables: firmness (FRM), titratable acidity (TA), weight loss (WL), total soluble solids (TSS), ripening index (MI), antioxidant activity (DPPH and ABTS), total phenolics (TP), and ascorbic acid (AA). Ellipses represent the variability associated with each UV-C radiation dose (0–5 kJ·m^−2^). (**B**–**F**) Individual biplots for each UV-C dose showing fruit progression during storage (numbers inside circles indicate storage days). Arrows indicate the contribution of each variable to sample separation within each treatment.

Principal component analysis (PCA) allowed the identification of joint variation patterns among the physicochemical and functional variables of seriguela fruits during storage (Figure 6). The first two principal components explained 84.7% of the total variability (PC1 = 61.1%; PC2 = 23.6%), demonstrating the ability of the model to represent the main data trends. This high explained variance indicates that the selected variables were sufficient to capture the major physiological changes occurring during fruit ripening and storage. In general, samples corresponding to the initial storage stages (0 and 1 day) were mainly associated with firmness (FRM) and DPPH values. Considering that the DPPH assay was expressed as EC_50_, higher values indicate lower antioxidant capacity, suggesting that freshly harvested fruits exhibited greater mechanical resistance but lower antioxidant activity compared with more advanced ripening stages. This distribution reflects the typical metabolic profile of early-stage fruits, characterized by structural integrity and lower activation of antioxidant defense systems. As storage progressed, a gradual displacement of the samples toward the positive direction of PC1 was observed, where variables such as soluble solids (TSS), total phenolics (TP), antioxidant activity determined by ABTS, and mass loss (WL) were grouped. These parameters are associated with ripening progression and metabolic changes occurring in the fruits. This shift indicates a coordinated transition from a structurally dominant state to a metabolically active state, in which biochemical transformations and oxidative processes become more pronounced. This pattern indicates that PC1 mainly represents a ripening gradient, separating fruits with characteristics typical of the early storage stage from those at more advanced stages of maturation. In contrast, PC2 appears to differentiate secondary variations among treatments, possibly related to the intensity of metabolic responses induced by UV-C radiation. Multivariate statistical approaches such as principal component analysis (PCA) have been widely applied in postharvest studies of tropical fruits to identify physiological patterns associated with ripening and storage conditions. Similar clustering patterns have been reported in studies evaluating metabolic and quality changes during storage of tropical fruits, where multivariate analyses helped distinguish metabolic gradients and treatment effects throughout the ripening process [23,34]. Thus, the PCA results reinforce that UV-C treatments influenced the metabolic trajectory of the fruits during storage, even when some individual variables did not show significant differences in univariate analyses.

Among the treatments, the doses of 2 and 4 kJ·m^−2^ showed a more concentrated distribution of samples throughout the storage days, suggesting lower physiological variation over time. In contrast, the control and the 5 kJ·m^−2^ treatment exhibited greater dispersion of points, indicating higher heterogeneity of physiological responses during storage. These results are consistent with the individual parameters evaluated, such as firmness and mass loss, and suggest that intermediate UV-C radiation doses may contribute to greater physiological stability of the fruits during storage. Principal component analysis revealed a clear metabolic gradient along PC1, separating variables associated with structural freshness (firmness and DPPH EC_50_) from those related to fruit ripening, including soluble solids, total phenolics, ABTS antioxidant activity, and mass loss. Interestingly, fruits treated with intermediate UV-C doses (2 and 4 kJ·m^−2^) were positioned between these two metabolic groupings, suggesting a moderate progression of ripening. This pattern indicates that intermediate UV-C doses may modulate metabolic activity, delaying ripening while maintaining physiological balance during storage.

**Figure 7:**
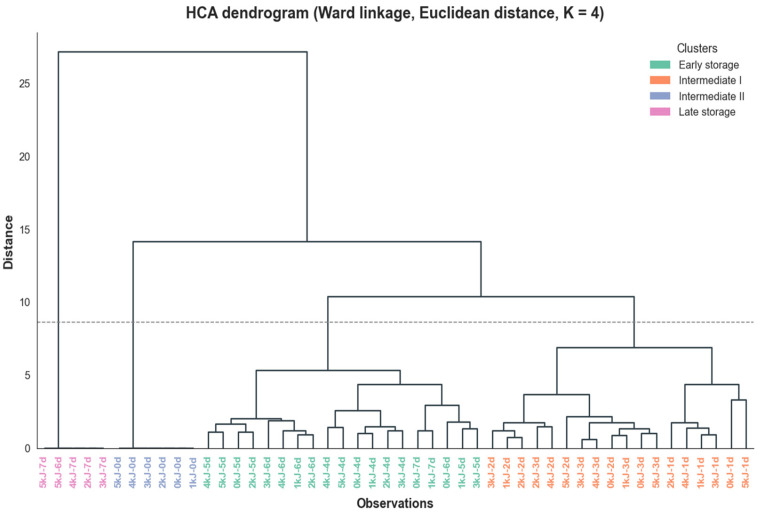
Hierarchical Cluster Analysis (HCA) dendrogram of UV-C-treated *Spondias purpurea* fruits during storage, based on physicochemical and functional quality variables (Ward linkage, Euclidean distance). The height of each branch represents the dissimilarity between samples, and the colors indicate distinct clusters with similar overall quality profiles. The dotted line indicates the cut-off distance used to define the clusters (K = 4).

The dendrogram obtained by hierarchical cluster analysis (HCA) (Figure 7) reinforces the patterns observed in the PCA, separating the samples into four main groups: Early storage, Intermediate I, Intermediate II, and Late storage. The Early storage group predominantly includes samples from the first days of storage (0–1 day) for all treatments, characterized by higher firmness (FRM) and higher DPPH values expressed as EC_50_, indicating fruits with greater structural integrity but lower antioxidant activity at this initial stage. The intermediate groups mainly include samples between 2 and 5 days of storage, associated with increases in soluble solids (TSS) and total phenolic compounds (TP), reflecting the progression of ripening and the intensification of fruit metabolic activity. The height at which the clusters merge, close to a Euclidean distance of 9, represents the overall level of dissimilarity among the groups, highlighting the distinction between early and advanced storage stages.

The Late storage group mainly comprised samples from the final storage days (6–7 days), with greater representation of the control (0 kJ·m^−2^) and the highest UV-C dose (5 kJ·m^−2^). In contrast, samples subjected to intermediate UV-C doses (1–4 kJ. m^−2^) remained predominantly grouped within the intermediate clusters, suggesting a slower progression of physiological changes during storage. This pattern, consistent with the results observed in the PCA, indicates that moderate UV-C doses may delay ripening progression, whereas the highest dose tends to exhibit behavior more similar to the control during the final stages of storage.

Overall, the results indicate that intermediate UV-C doses between 2 and 4 kJ·m^−2^ provided the most balanced response in terms of postharvest quality preservation. These treatments reduced mass loss, delayed softening, and maintained color stability while promoting higher levels of phenolic compounds and antioxidant activity during storage. In contrast, the highest dose (5 kJ·m^−2^) tended to accelerate physiological changes and shorten the storage period in some cases, suggesting that excessive UV-C exposure may induce stress responses that negatively affect fruit stability. Therefore, moderate UV-C doses appear to stimulate beneficial metabolic responses without causing tissue damage.

## 4. Conclusions

Among the evaluated treatments, UV-C doses between 2 and 4 kJ·m^−2^ showed the most favorable effects on the postharvest quality of seriguela fruits. These doses reduced mass loss, delayed fruit softening, and preserved color stability while promoting the accumulation of phenolic compounds and antioxidant activity. In contrast, the highest dose (5 kJ·m^−2^) showed behavior similar to or less stable than the control in some parameters, suggesting that excessive UV-C exposure may accelerate physiological stress. Therefore, intermediate UV-C doses represent the most suitable range for maintaining the physicochemical and functional quality of seriguela during ambient storage. Future studies should explore the combination of UV-C radiation with other postharvest technologies, such as edible coatings and modified atmosphere storage, as well as evaluate its effects under different storage conditions, including refrigeration, to allow comparison with standard commercial practices. In addition, physiological, enzymatic, microbiological, and sensory analyses, including respiration rate, ethylene production, cell wall-related enzymes, microbial load assessment, and consumer acceptance attributes (color, aroma, taste, and texture), should be investigated to better elucidate the mechanisms and overall effectiveness of UV-C treatment during storage.

## Data Availability

The original contributions presented in this study are included in the article. Further inquiries can be directed to the corresponding author.
